# Supplementary material for: Plant-Derived Nutraceuticals Involved in Body Weight Control by Modulating Gene Expression
Source: Plants (Basel). 2023 Jun 11;12(12):2273. doi: 10.3390/plants12122273 (PMC10302664; doi:10.3390/plants12122273)
Supplement: Supplementary file 1 [file plants-12-02273-s001.zip › plants-2420329-supplementary.pdf]

Supplementary Material

Table S1. Epigenetic modifications induced by nutraceuticals in relation to obesity

Nutraceutical	Model	Effects	Targeted genes or pathways	Gut microbiota	miR	Study
**Resveratrol**	Humans<br>In vitro and animal models	Modulation of mitochondrial activity<br>Glucose homeostasis<br>decrease adipogenesis<br>increase thermogenesis<br>Reducing of fat acumulation<br>Stimulation of browning process<br>Reduction of body weight<br>Reduction of subcutaneous adipose tissue<br>Protection against sarcopenic obesity	AMPK ↑<br>SIRT-1 ↑<br>CCAAT-C/EBPα ↓<br>UCP1 ↑<br>FNDC5 ↑<br>SREBPs ↓<br>FIAF ↑<br>LPL ↓<br>PKA/LKB1/AMPK ↑	*Bacteroidetes/Firmicutes ratio* ↑<br>*Lactobacillus* ↑<br>*Bifidobacterium* ↑<br>*Enterococcus faecalis* ↓	miR-211-3p ↑<br>miR-1224 ↑<br>miR-539-5p ↑<br>miR-511-3p ↓	61, 63-70, 75, 78
**Curcumin**	Humans<br>In vitro and animal models	Antiadipogenic<br>Anti-inflammatory<br>Increase thermogenesis and energy expenditure<br>Reduces insulin resistance	C/EBPα ↓<br>KLF5 ↓<br>PPARγ ↓<br>aP2 ↓<br>MAPK ↑<br>STAT3 ↓<br>IL-1β ↓<br>UCP1↑<br>STAT3 ↓	*Bacteroidaceae* ↑<br>*Prevotellaceae* ↓<br>*Rikenellaceae* ↑<br>*Anaerotruncus* ↓<br>*Exiguobacterium* ↑<br>*Helicobacter* ↓<br>*Shewanella* ↑<br>*Serratia* ↑<br>*Lactococcus* ↑<br>*Turicibacter* ↑<br>*Parasutterella genera* ↑	miR-17-5p ↑	82, 83, 85-101
**Ginger**	Humans<br>In vitro and animal models	Antiadipogenic<br>Anti-inflammatory and hypoglycemic<br>Reduction of hepatic lipid accumulation<br>Increase thermogenesis and energy expenditure, reduce body weight and waist circumferences	C/EBPα ↓<br>PPARγ ↓<br>aP2 ↓<br>FAS ↓<br>AKT/GSK3 ↓<br>TNFα ↓<br>IL6 ↓<br>FGF2 ↑<br>CPT1 ↑<br>ACOX1 ↑<br>UCP1 ↑	*Firmicutes/Bacteroidetes ratio* ↑<br>*Proteobacteria* ↑<br>*Ruminococcus* ↓	miR-21 ↓<br>miR-132 ↓	115-129 133, 136, 139
**Epigallocatechin-3-gallate (EGCG)**	Humans<br>In vitro and animal models	Antiobesity<br>Increases lipolysis and lipid oxidation	AMPK ↑<br>HSL ↑	*Bacteroides* ↑<br>*Parasutterella* ↑	miR-1297 ↓<br>miR-373-3p ↓<br>miR-192-5p ↓	

					miR-1266-5p ↓<br>miR-595 ↓	
		Enhances basal metabolism	ATGL ↑	*Allobaculum* ↓		
		Decreases weight gain	ACOD2 ↑	*Roseburia* ↓		
		Decreases adipose tissue weight	PPARγ ↑	*Erysipelotrichacea* ↓		
		Decreases calorie intake	MCAD ↑	*Lachnospiraceae* ↓		
			UCP3 ↑	*Ruminococcaceae* ↓		
			NRF1 ↑	*Anaerotruncus* ↓		
			COX-2 ↓	*Odoribacter* ↓		
		Suppresses liver inflammation	iNOS ↓	*Enterorhadus* ↓		142-148<br>153-157
			DRP1 ↓	*Lachnospiracea* ↓		
		Amelioration of muscle autophagy in diabetes	Beclin1 ↓	*Akkermansia* ↑		
				Christensenellaceae ↑		
			PPARGα ↑	*Bifidobacterium* ↑		
		Improvement in insulin sensitivity and lipid profile		*Fusobacterium varium* ↓		
			GLUT4 ↑			
			LPL ↑	*Enterobacteriaceae* ↓		
				Bilophila ↓		
**Capsaicin**	Humans<br>In vitro and animal models	Antiobesity<br>induce body weight reduction	LEP ↓<br>PPARγ ↓	*Akkermansia* ↑<br>*Bacteroides*↑	mmu-let-7a-5p ↑<br>mmu-let-7d-5p ↑<br>mmu-let7b-3p ↑	165-179
			C/EBP-α ↓<br>PPARα ↑	*Prevotella* ↑<br>*Allobaculum* ↑		
		Improves lipolysis in adipocytes				
			PGC-1α ↑<br>TRPV-1 ↓	*Odoribacter* ↑<br>*Coprococcus* ↑		
		Increases energy expenditure				
		Increases satiety	ADIPOQ ↑<br>UCP1 ↑	*SCFAs* ↑<br>Acetate concentrations ↑		
		Decreases the desire to eat	SIRT-1 ↑<br>BMP8b ↑	Propionate concentrations ↑		
		Improves glucose intolerance	PGC-1α ↑			
		Increases thermogenesis and improves cholesterol level	BDNF ↑<br>PRMD16 ↑	*Escherichia* ↓<br>*Desulfovibrio* ↓		
			FOXC2 ↑<br>NCOA1 ↑<br>DIO2 ↑	*Sutterella* ↓<br>*Helicobacter* ↓		
		Conversion of white/beige cells into brown adipocites				
			SIRT1 ↑			
**Caffeine**	Humans	Anti-obesity	PI3K/AKT activity ↓	*Firmicutes* /*Bacteroidetes ratio* ↑		191-199<br>200, 201
	In vitro and animal models	Reduces food intake	PPARγ ↓			
		Increases energy expenditure	UCP1 ↑	*Bifidobacterium spp* ↑<br>*Prevotella* ↑		
		Anti-adipogenic<br>Reduces adipocytes number	FAS ↓<br>CCAAT-C/EBPα ↓	*Porphyromonas* ↑		
		Improves dyslipidemia<br>Anti-inflammatory	C/EBPβ ↓<br>SREBP-1<br>TNFα ↓<br>MCP-1 ↓<br>IL-6 ↓			
